# Supplementary figures and images for: HLA Upregulation During Dengue Virus Infection Suppresses the Natural Killer Cell Response
Source: Front Cell Infect Microbiol. 2019 Jul 23;9:268. doi: 10.3389/fcimb.2019.00268 (PMC6663972; doi:10.3389/fcimb.2019.00268)

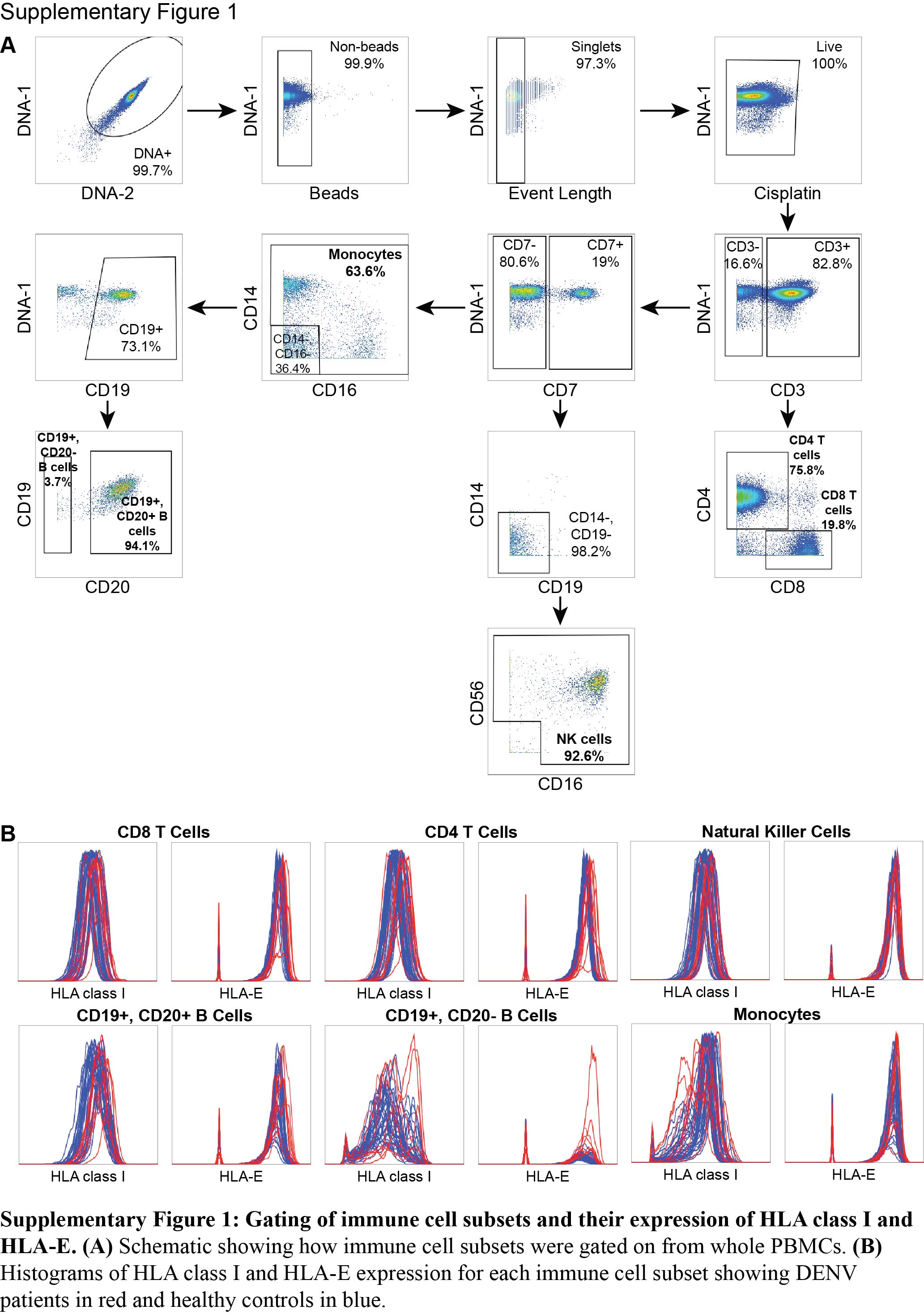

Supplement: Supplementary file 2 [file Image_1.JPEG]

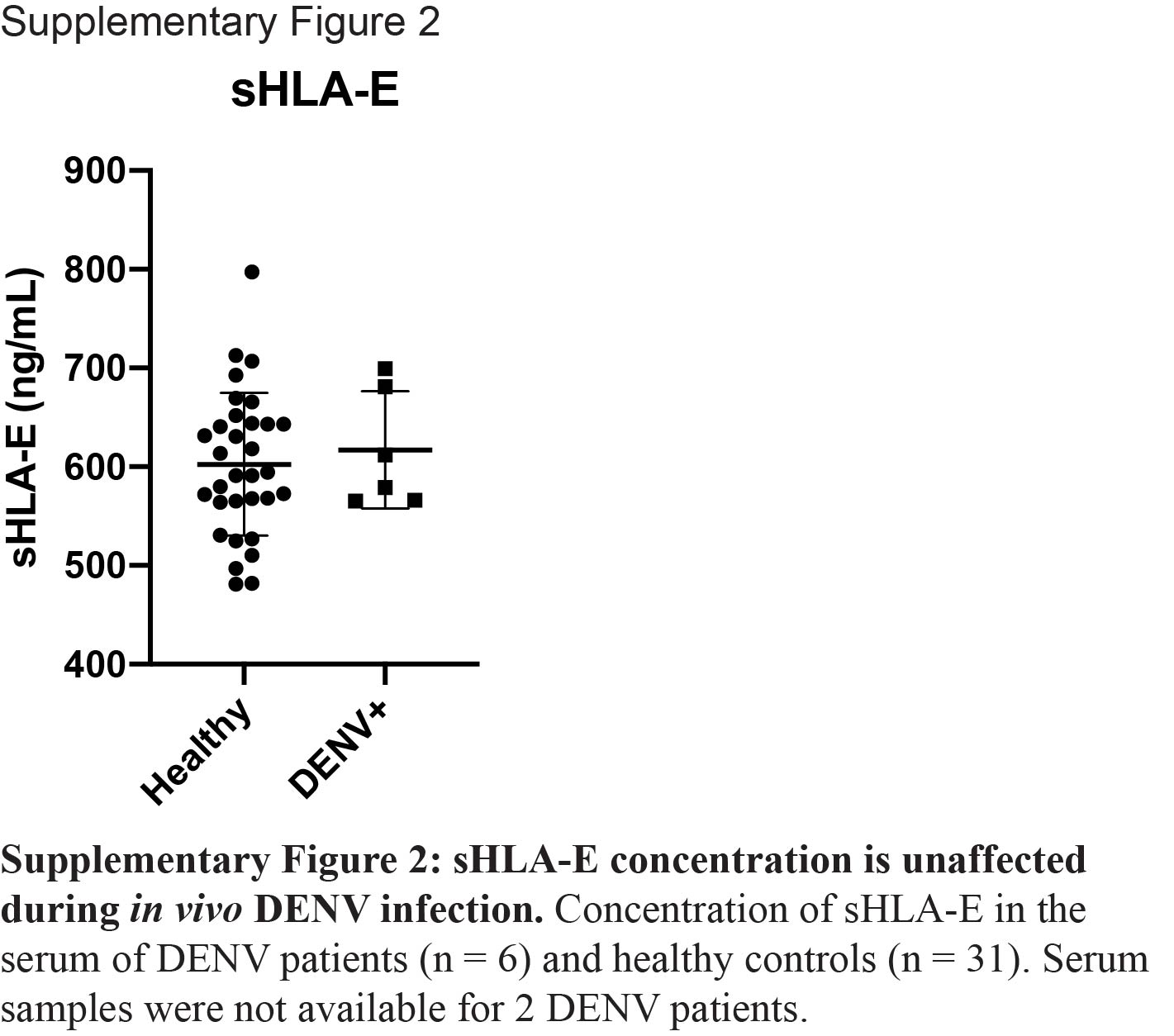

Supplement: Supplementary file 3 [file Image_2.JPEG]

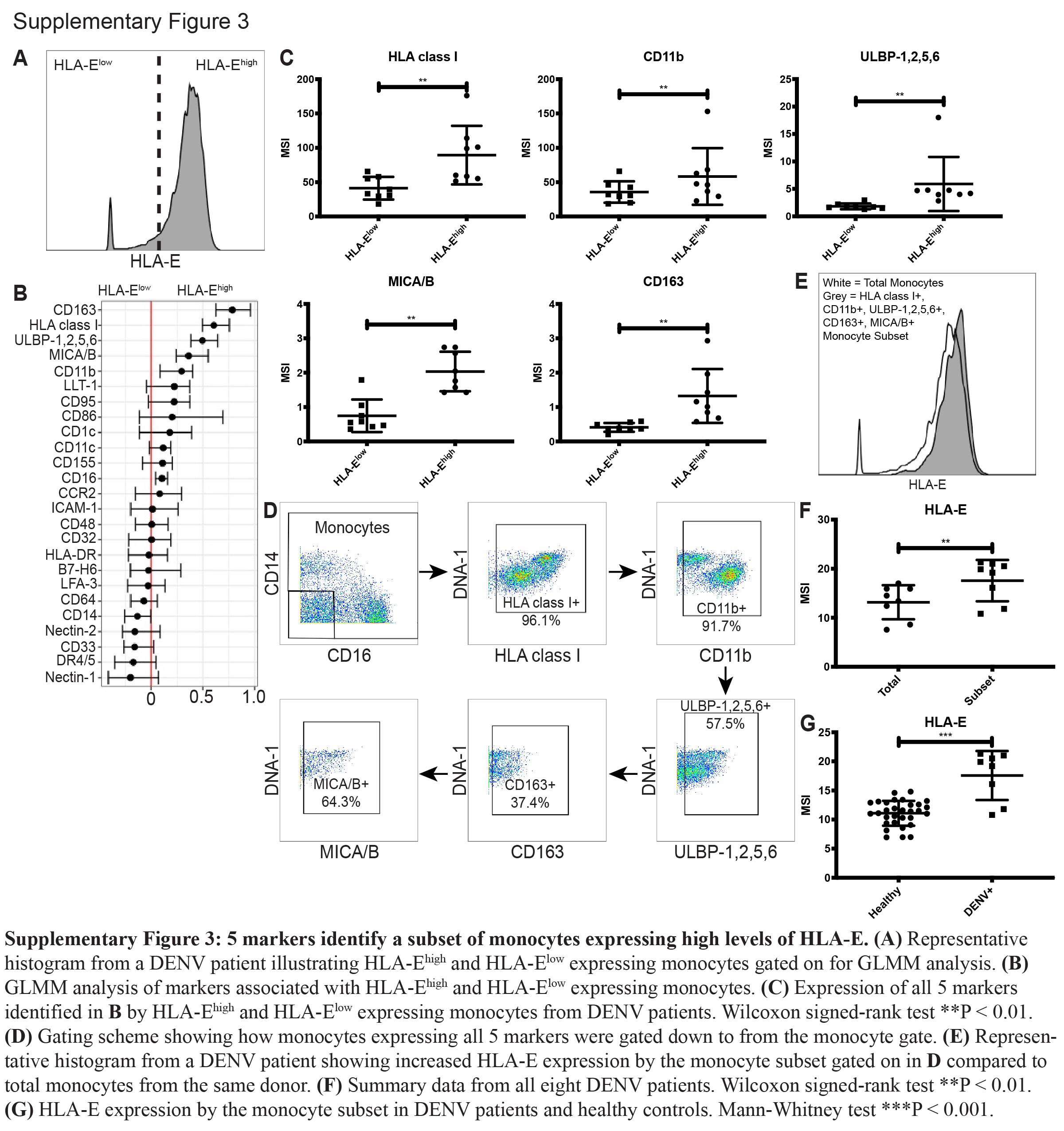

Supplement: Supplementary file 4 [file Image_3.jpg]

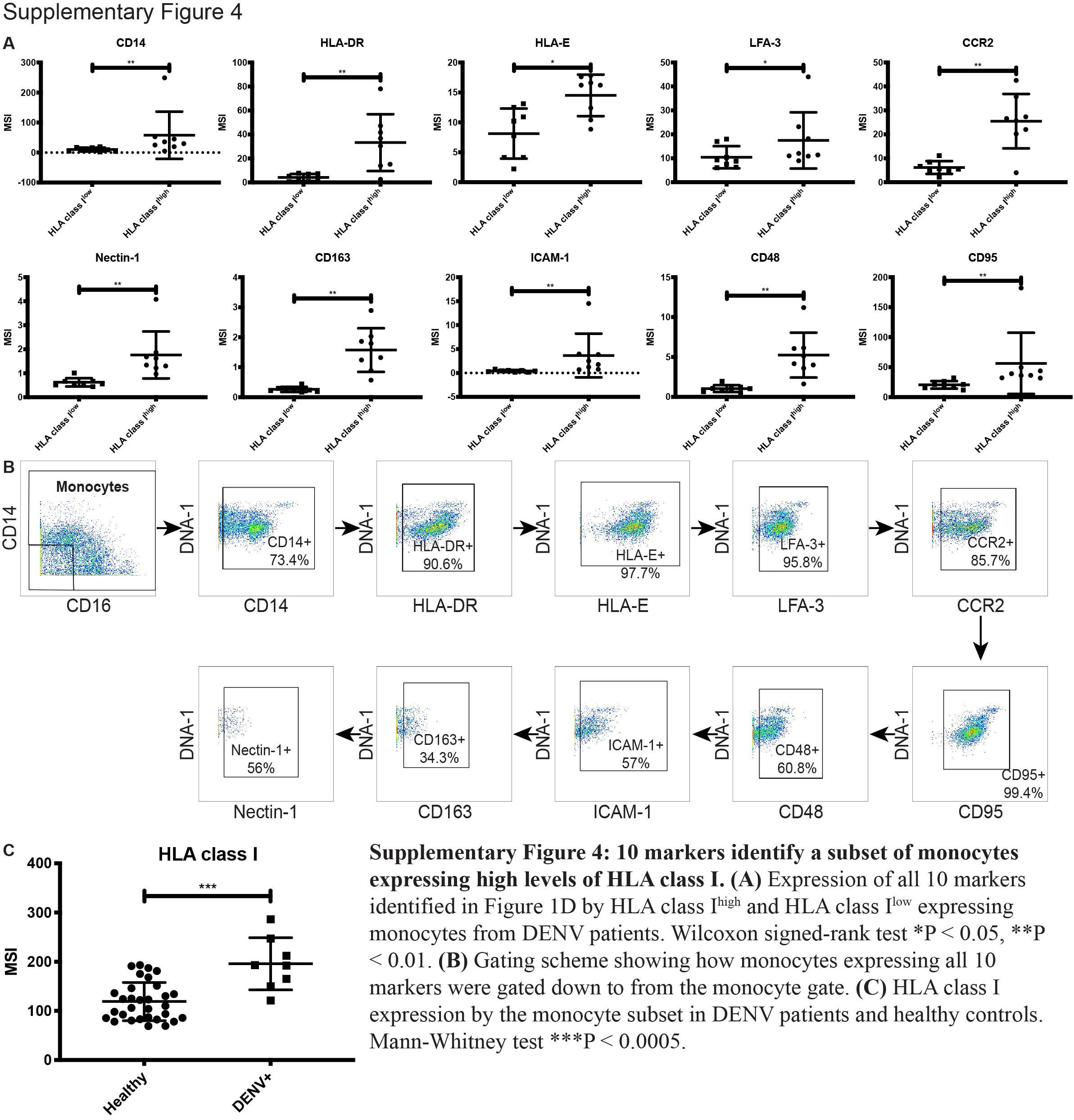

Supplement: Supplementary file 5 [file Image_4.JPEG]

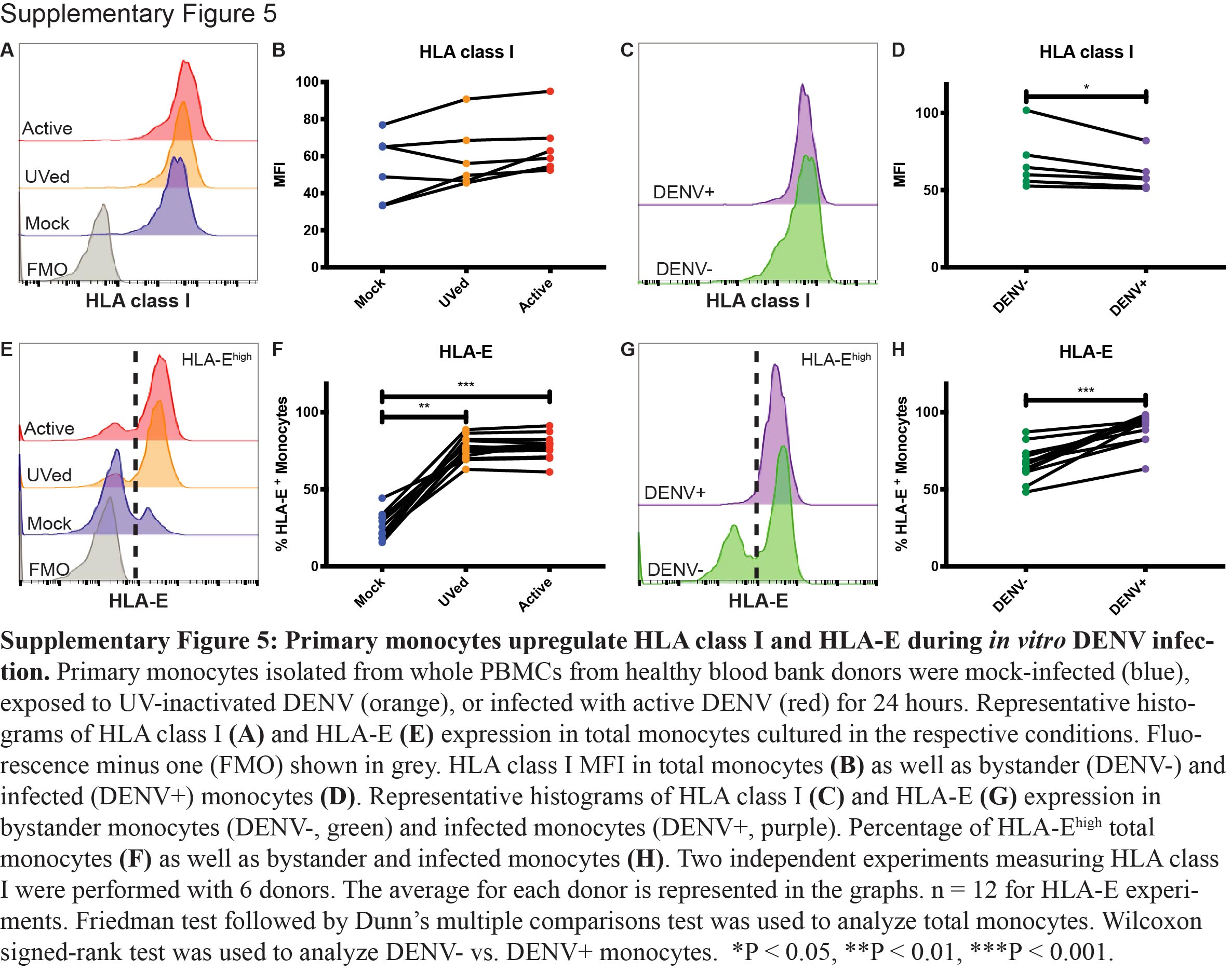

Supplement: Supplementary file 6 [file Image_5.jpg]
